# Molecular characterization and epidemiological investigation of colistin resistance in carbapenem-resistant *Klebsiella pneumoniae* in a tertiary care hospital in Tehran, Iran

**DOI:** 10.1186/s12866-024-03376-4

**Published:** 2024-06-28

**Authors:** Neda Razavi Davoodi, Neda Soleimani, Seyed Masoud Hosseini, Marjan Rahnamaye-Farzami

**Affiliations:** 1 Department of Microbiology and Microbial Biotechnology, Faculty of Life Sciences and Biotechnology, Shahid Beheshti University, Tehran, Iran; 2 Department of Microbiology, Research Center of Health Reference Laboratory, Ministry of Health and Medical Education, Tehran, Iran; 3 Department of Microbiology, Reference Health Laboratory Research Center, Ministry of Health and Medical Education, Tehran, Iran

**Keywords:** *Klebsiella pneumoniae*, Carbapenemases, *Mcr* genes, Colistin, PFGE

## Abstract

**Background:**

Carbapenemase-producing *Klebsiella pneumoniae* (CRKP) presents a significant challenge to antimicrobial therapy, especially when compounded by resistance to colistin. The objective of this study was to explore molecular epidemiological insights into strains of clinical *K. pneumoniae* that produce carbapenemases and exhibit resistance to colistin. Eighty clinical isolates of CRKP were obtained from Milad Hospital in Tehran, Iran. Antimicrobial susceptibility and colistin broth disk elution were determined. PCR assays were conducted to examine the prevalence of resistance-associated genes, including *bla*_*KPC*_, *bla*_*IMP*_, *bla*_*VIM*_, *bla*_*OXA−48*_, *bla*_*NDM*_ and *mcr-*1 to -10. Molecular typing (PFGE) was used to assess their spread.

**Results:**

Colistin resistance was observed in 27 isolates (33.7%) using the Broth Disk Elution method. Among positive isolates for carbapenemase genes, the most frequent gene was *bla*_*OXA−48*_, identified in 36 strains (45%). The *mcr-1* gene was detected in 3.7% of the obtained isolates, with none of the other of the other *mcr* genes detected in the studied isolates.

**Conclusion:**

To stop the spread of resistant *K. pneumoniae* and prevent the evolution of *mcr* genes, it is imperative to enhance surveillance, adhere rigorously to infection prevention protocols, and implement antibiotic stewardship practices.

**Supplementary Information:**

The online version contains supplementary material available at 10.1186/s12866-024-03376-4.

## Background

*Klebsiella pneumoniae* is a Gram-negative bacterium (GNB) and a significant pathogen in nosocomial infections, particularly in intensive care units (ICUs). It is responsible for severe infections such as urinary tract infections (UTIs), pneumonia, bacteremia, neonatal meningitis, and pyogenic liver abscesses. Over recent years, the emergence of multidrug-resistant (MDR) and extensively drug-resistant (XDR) *K. pneumoniae*, along with the absence of new antibiotics capable of combating them, has become a serious global issue [1].

Over the past several years, carbapenem antibiotics have served as effective last-line treatments for infections caused by MDR *Enterobacterales*. However, the recent emergence of carbapenemase-producing *Enterobacterales*, particularly *K. pneumoniae*, has been associated with higher mortality rates (up to 40–50%), particularly in bloodstream infections (BSIs) and ICU admissions. This has led to the consideration of colistin as one of the last and most effective options for treating carbapenem-resistant *K. pneumoniae* (CRKP) infections. Nevertheless, the observed increase in resistance to this antibiotic indicates a worrisome trend that undermines the efficacy of this once highly efficient treatment option [2].

In 2015, the discovery of the mobilized colistin resistance (*mcr*) gene marked a significant development, as it was found to confer unique colistin resistance (CLR) in *Enterobacterales* isolated. Subsequent studies have identified *Enterobacterales* carrying *mcr* genes worldwide, spanning across livestock, food, and humans populations, suggesting the potential for horizontal transmission of colistin resistance. This has raised concerns about the emergence of pandrug resistance in *Enterobacterales*. Therefore, it remains crucial to continuously and precisely monitor the emergence and spread of *mcr* genes among bacteria. Variants ranging from *mcr-1* to *mcr-10* have been documented to date [3]. The *mcr* gene mediates resistance to colistin by encoding an enzyme that adds phosphoryl ethanolamine to the lipid A present in the cell membrane of gram-negative bacteria, resulting in an altered lipid A with much lower affinity for colistin [4].

A systematic review and meta-analysis on the prevalence of colistin resistance among *K. pneumoniae* isolates in Iran revealed that the pooled prevalence of CLR in clinical isolates was 6.9% [5]. However, the rate of CRKP was reported to be over 73% in various studies [6, 7]. The development of reliable and cost-effective techniques for detecting colistin resistance is essential. Simner and colleagues introduced the Colistin Broth Disk Elution method, which utilizes colistin disks as a source of these antibiotics [8].

Additionally, it is imperative to develop and execute appropriate studies to bolster antimicrobial resistance programs and furnish additional data to inform evidence-based policy decisions [9]. In this regard, molecular characterization and genotyping of isolated strains of *K. pneumoniae* from hospital patients, along with determining the resistance mechanisms in these isolates, would be a helpful survey. This effort becomes even more valuable if complemented by an effective tool for monitoring and controlling the spread of epidemic-associated clones between different hospital environments and investigating the primary sources of bacterial contamination. Among the array of molecular methods available, pulsed-field gel electrophoresis (PFGE) is currently recognized as a suitable approach for typing *K. pneumoniae* isolates and tracing their spread [10].

The objective of this study was to investigate the molecular mechanisms underlyingof colistin and carbapenem resistance in a collection of XDR CRKP isolated obtained from clinical specimens in Tehran, Iran. Additionally, the study aimed to describe the clonal relationships among these isolates.

## Materials and methods

### Bacterial isolates

Between August 2020 and February 2021, Milad Hospital in Tehran isolated a total of 80 non-duplicate strains of CRKP from clinical samples of both inpatients and outpatients. These strains exhibited resistance to either meropenem or imipenem during initial screening. Milad Hospital is a tertiary care facility with 1,000 beds, affiliated with the Social Assurance Organization. All isolates were obtained from clinical samples, including urine, blood, sputum, and tracheal aspirate. The bacterial isolates were reidentified as *K. pneumoniae* using biochemical methods including oxidase, sugar fermentation, IMViC, Kliger’s iron agar, nitrate reduction, and motility tests [11].

### Assessment of antimicrobial susceptibility using the disk diffusion method

The susceptibility of CRKP isolates to 11 antibiotics specified by CLSI M100-Ed31, including ceftriaxone (30 µg), tobramycin (10 µg), piperacillin-tazobactam (10 µg), amikacin (30 µg), levofloxacin (5 µg), ceftazidime (30 µg), ciprofloxacin (5 µg), gentamicin (10 µg), meropenem (10 µg), imipenem (10 µg), and cefepime (30 µg) (MAST DISCS™ ID, UK), was determined using the standard disk diffusion method [12]. The results were interpreted according to the recommended criteria, with standard strains *Escherichia coli* ATCC 25,922 and *Pseudomonas aeruginosa* ATCC 27,853, were used as quality control strains for susceptibility testing. In accordance with the guidelines of the Centers for Disease Control and Prevention in the United States and the European Centre for Disease Prevention and Control, All isolates were identified as XDR These *K. pneumoniae* isolates demonstrated resistance to at least one agent in all antimicrobial categories, with the exception of two or fewer, indicating susceptibility to only one or two categories [13].

### Determination of Minimum Inhibitory Concentration (MIC) against Colistin

MIC against colistin was determined using colistin (10 µg) discs (Neo-Sensitabs™, Rosco, Denmark) in the assay. The colistin broth disk elution method described in CLSI guidelines was used for the antimicrobial susceptibility test [12].

### Detection of *mcr-1* to *mcr-10* genes by PCR

Genomic DNA was extracted using the Genomic DNA Purification Kit (QIAGEN^®^ Kit, QIAGEN, Germantown, MD, USA) following the manufacturer’s instructions. The presence of isolates carrying *mcr* genes was determined through PCR amplification and subsequently confirmed by sequencing. DNA samples from *E. coli* SHP45 and *E. coli* KP37, known to carry the *mcr-1* and *mcr-2* genes, respectively, were utilized as positive controls in the assay. Additionally, genomic DNA from colistin-susceptible *E. coli* ATCC 25,922 served as the negative control. These strains were sourced from the Iranian Reference Health Laboratory. The primers for *mcr* genes are listed in Table 1 [14–18].

**Table 1:** The list of primers, annealing temperatures, and expected amplicon sizes for molecular detection of *mcr* genes and carbapenemases-producing *K. pneumoniae* isolates

Gene	Sequence	TM (°C)	Amplicon size (bp)	References
*mcr-1-*F	5-AGTCCGTTTGTTCTTGTGGC-3	55	320	[14]
*mcr-1-*R	5-AGATCCTTGGTCTCGGCTTG-3			
*mcr-2-*F	5-CAAGTGTGTTGGTCGCAGTT-3	58	715	[14]
*mcr-2-*R	5-TCTAGCCCGACAAGCATACC-3			
*mcr-3-*F	5-TTGGCACTGTATTTTGCATTT-3	50	542	[15]
*mcr-3-*R	5-TTAACGAAATTGGCTGGAACA-3			
*mcr-4-*F	5- GATCCGAAGCTGTGTTCTG-3	59	426	[16]
*mcr-4-*R	5- GCCAGCATTGGTACGCTAGT-3			
*mcr-5-*F	5- GGTTGGCCGAGAAGATAACA-3	59	522	[16]
*mcr-5-*R	5- ATGTTGCCAGAAGGTCCAAC-3			
*mcr-6-*F	5- AGCTATGTCAATCCCGTGAT − 3	55	252	[17]
*mcr-6-*R	5- ATTGGCTAGGTTGTCAATC − 3			
*mcr-7-*F	5- GTCAGTTACGCCATGCTCAA-3	59	791	[16]
*mcr-7-*R	5- TTCTTGTCGCAGAACTGTGG-3			
*mcr-8-*F	5- AAACTGAACCCGGTACAACG-3	59	943	[16]
*mcr-8-*R	5- GCCATAGCACCTCAACACCT-3			
*mcr-9-*F	5- GCGGTTGTAAAGGCGTATGT-3	59	635	[16]
*mcr-9-*R	5- CAAATCGCGGTCAGGATTAT-3			
*mcr-10-*F	5- GCAATAACCCGACGCTGAAC-3	53	133	[18]
*mcr-10-*R	5- GTAACGCGCCTTGCATCATC-3			
*bla*_*KPC*_*-*F	5-CGTCTAGTTCTGCTGTCTTG-3	55	798	[19]
*bla*_*KPC*_*-* R	5-CTTGTCATCCTTGTTAGGCG-3			
*bla*_*VIM*_*-*F	5- GATGGTGTTTGGTCGCATA-3	57	390	[19]
*bla*_*VIM*_ *-R*	5- CGAATGCGCAGCACCAG-3			
*bla*_*IMP*_ *-F*	5- GGAATAGAGTGGCTTAAYTC-3	57	232	[19]
*bla*_*IMP*_ *-R*	5- TCGGTTTAAYAAAACAACCACC-3			
*bla*_*NDM*_ *-F*	5-GGTTTGGCGATCTGGTTTTC-3	52	621	[19]
*bla*_*NDM*_ *-R*	5- CGGAATGGCTCATCACGATC-3			
*bla*_*OXA−48*_ *-F*	5-GCGTGGTTAAGGATGAACAC-3	55	438	[19]
*bla*_*OXA−48*_ *-R*	5- CATCAAGTTCAACCCAACCG-3			

### Detection of carbapenemase-encoding genes

Multiplex PCR was employed to detect *bla*_*NDM*_, *bla*_*IMP*_, *bla*_*VIM*_, *bla*_*KPC*_, and *bla*_*OXA-48*_. The positive and negative controls for PCR experiments were *K. pneumoniae* ATCC strain BAA-1705 and *K. pneumoniae* ATCC BAA-1706, respectively. PCR experiments used the specific oligonucleotide primers listed in Table 1 [19].

### Genotyping with PFGE

All isolates were typed using a PFGE technique following the PulseNet Standardized Laboratory Protocol [13]. The genomic DNA from *Salmonella enterica* serotype Braenderup H9812 digested with XbaI (Thermo Fisher Scientific, USA) served as a molecular size marker. DNA banding patterns were analyzed using BioNumerics software, version 6.6 (Applied-Maths, Sint-Martens-Latem, Belgium). The analysis employed the Dice correlation coefficient and the UPGMA (unweighted pair group method using an arithmetic mean algorithm) method with a band tolerance and optimization set at 1.5%.By comparing the PFGE results and applying the criteria of Tenover et al. based on the number of observed band differences, a cutoff value of 80% similarity overall was set for related isolates [20].

## Results

### Sample Collection

**Fig. 1:**
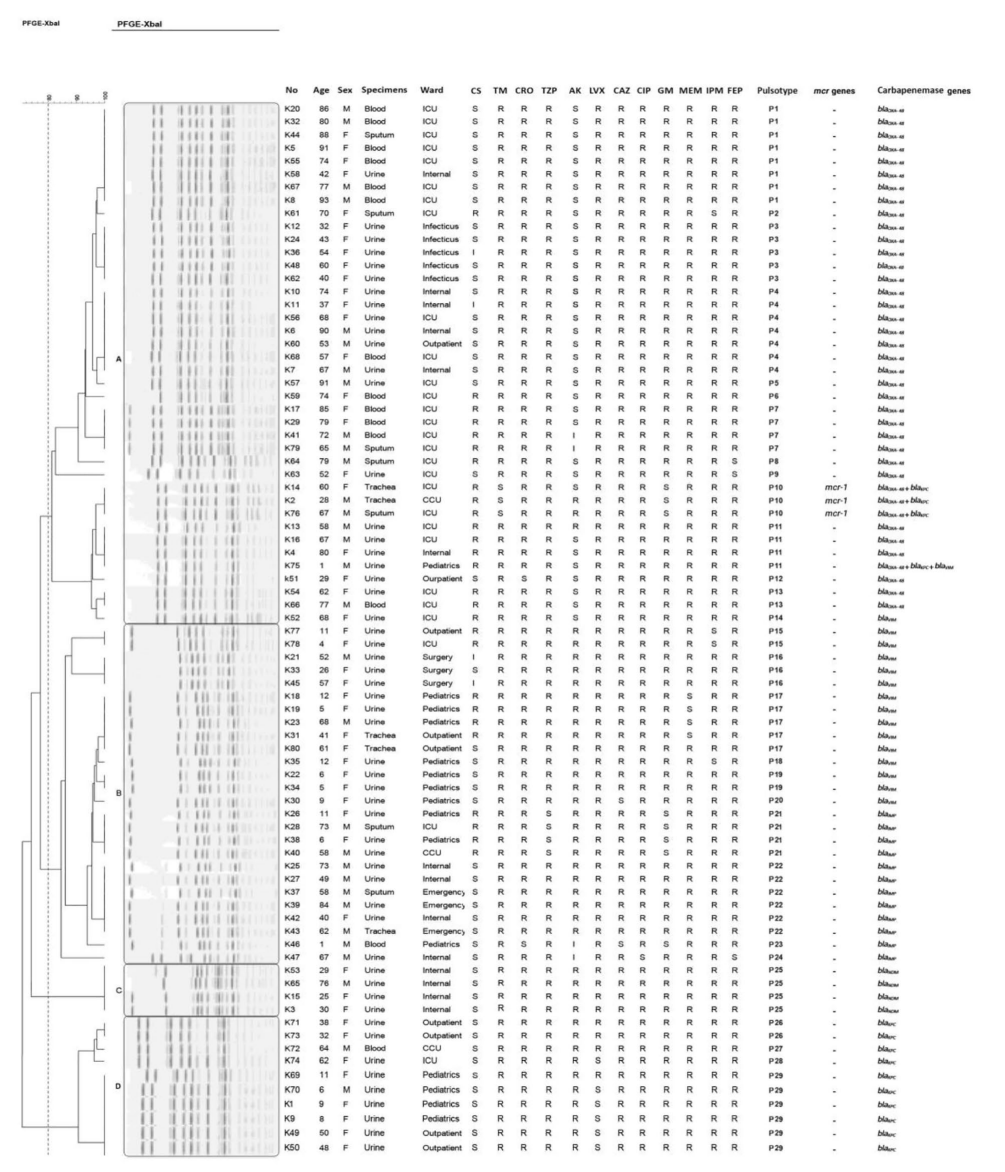
The clustering results of the 80 carbapenem-resistant *K. pneumoniae* (CRKP) isolates, determined by PFGE patterns following digestion with the XbaI enzyme, were correlated with the presence of *mcr*, Carbapenemase genes and antibiotic resistance profiles. The information of strain is listed to the right of the patterns. The four PFGE cluster (A), (B), (C), and (D) are represented by rectangles. Full-length gels are presented in Supplementary Figs. 1, 2, 3, 4 and 5

Out of the 80 CRKP isolates collected from various wards of Milad Hospital in Tehran, Iran, the majority of isolates were from urine (54, 67.5%), followed by blood samples (14, 17.5%), and both sputum and tracheal aspirate each accounted for 6 isolates (7.5%). The prevalence of CRKP in different hospital wards is depicted in Fig. 1. The ICU ward had the highest rate, with 25 isolates (31.2%), while surgery and emergency wards each had three isolates (3%), showing the lowest rates, respectively. Additionally, 61.2% (*n* = 49) of the isolates were from female patients, while 38.8% (*n* = 31) were from male patients. The mean age of patients with these isolates was 49.61 ± 3 (between 1 and 93 years). Among the 80 patients, the majority, 71, were inpatients, with only 9 being outpatients.

### Antimicrobial susceptibility pattern of *K. pneumonia*

**Fig. 2:**
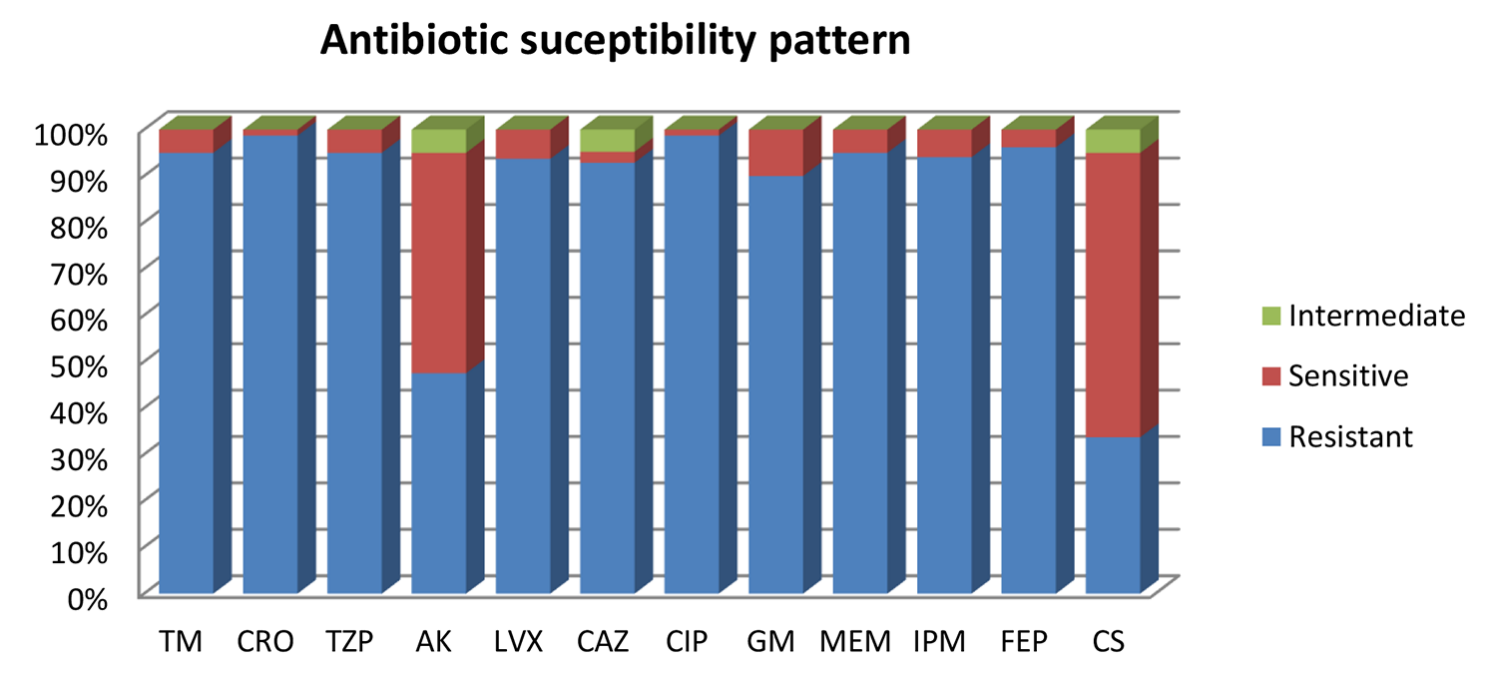
Antimicrobial resistance pattern of 80 *K. pneumoniae*. TM: Tobramycin, CRO: Ceftriaxone, TZP: Piperacillin/tazobactam, AK: Amikacin, LVX: Levofloxacin, CAZ: Ceftazidime, CIP: Ciprofloxacin, GM: Gentamicin, MEM: Meropenem, IPM: Imipenem, FEP: Cefepime, CS: Colistin

*K. pneumoniae* isolates exhibited a high resistance rate to ceftriaxone, ciprofloxacin (98.7%). The lowest resistance rates of all these isolates were observed with amikacin (47.5%). Resistance to other antibiotics was observed above 90%, as shown in Fig. 2. Among the 80 isolates, 33.7% (*n* = 27) were identified as CLR by colistin disk elution method.

### Detection of *mcr* genes

**Fig. 3:**
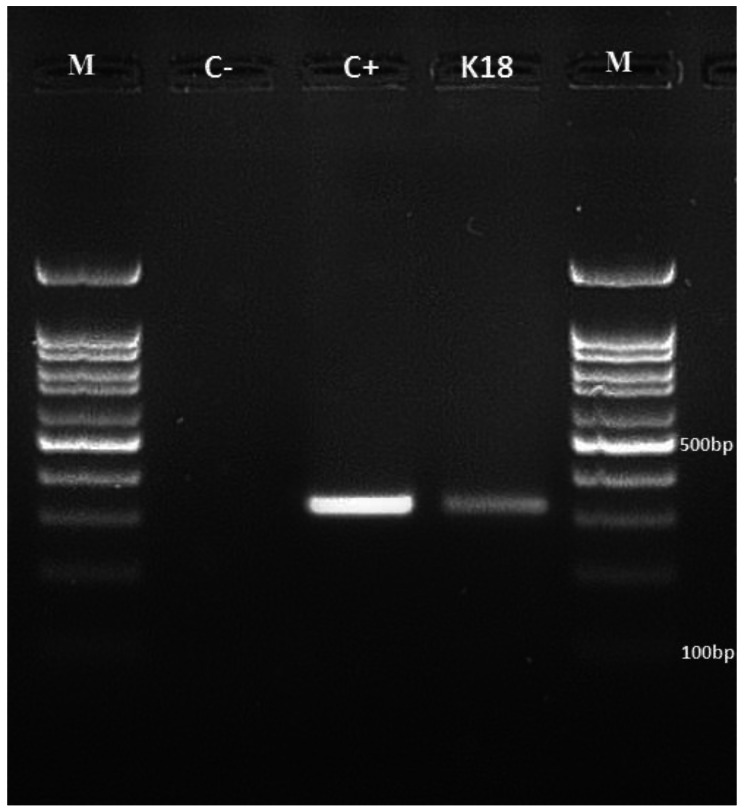
The electrophoresis analysis using (2%) agarose gel for the detection of *mcr − 1* genes, and the M: DNA ladder 100 bp; lanes (1) is a negative control, lanes (2) is a positive control, lanes (3): a *K. pneumoniae* clinical isolate

In this experiment, we used multiplex PCR screening to determine the prevalence of the *mcr-1* to *mcr-10* genes among the clinical *K. pneumoniae* isolates (Fig. 3). The *mcr-1* gene was detected in 3.7% (3 out of 80) of the obtained isolates. None of the studied isolates were found to carry the *mcr-2* to *mcr-10* genes. Table 2 indicates that there were no significant correlations between the CLR isolates and the presence *mcr-1* genes.

**Table 2:** The frequency of the *mcr-1* gene among phenotypically colistin-resistant isolates (*N* = 27)

Gene	Number	Percentage	*P* value
*mcr-1*	3/27	9%	> 0.05

### Molecular analysis of carbapenemase genes

**Fig. 4:**
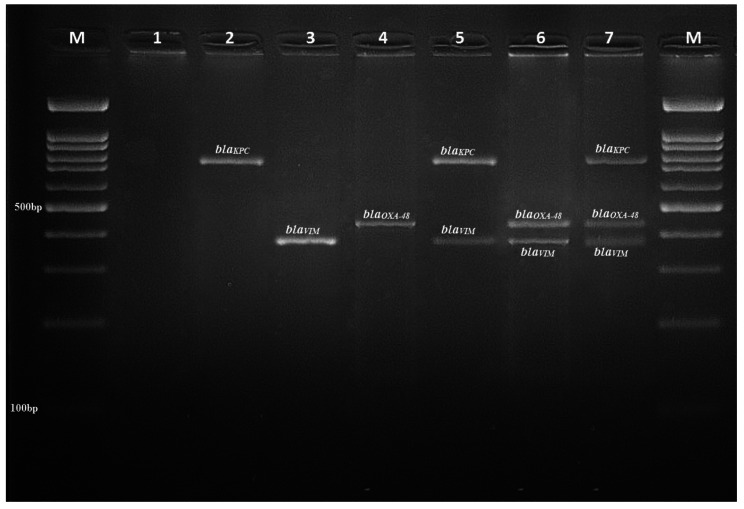
PCR results for carbapenemase-encoding genes; M: DNA ladder 100 bp, lanes (1) is a negative control, lanes (2) is a positive for *bla*_*KPC*_ (798 bp), lanes (3): positive for *bla*_*VIM*_ (390 bp), lanes (4): positive for *bla*_*OXA−48*_ (438 bp), lane (5): positive for *bla*_*IMP*_, lane (6): positive for *bla*_*NDM*_, lane (7): positive for *bla*_*IMP*_ and *bla*_*NDM*_

The isolates were examined by multiplex PCR for *bla*_*OXA-48*_, *bla*_*VIM*_, *bla*_*KPC*_, *bla*_*IMP*_, and *bla*_*NDM*_, and confirmed by sequencing (Fig. 4). The frequency of carbapenemase genes is displayed in Table 3. The gene encoding the OXA-48 enzyme was the most prevalent among the studied isolates and was identified in 36 strains (45%). It was followed by *bla*_*VIM*_, *bla*_*IMP*_, *bla*_*KPC*_, and *bla*_*NDM*_, in 14 (17.5%), 12 (15%), 10 (12.5%), and 4 (5%) strains, respectively. Additionally, co-existence of *bla*_*OXA-48*_ and *bla*_*KPC*_, and *bla*_*OXA-48*_, *bla*_*KPC*_, and *bla*_*VIM*_ genes were observed in 3 strains (3%) and 1 strain (1.2%), respectively. Furthermore, the results indicated no significant correlations between the CLR isolates and the detected carbapenemase genes.

**Table 3:** Prevalence of carbapenemase genes among isolates (*N* = 80)

Gene	Number	Percentage
*bla*_*OXA− 48*_	36	45%
*bla*_*VIM*_ *bla*_*IMP*_	16 12	17.5% 15%
*bla*_*KPC*_ *bla*_*NDM*_	10 4	12.5% 5%
*bla*_*VIM+*_ *bla*_*KPC*_	3	3%
*bla*_*OXA− 48+*_ *bla*_*KPC*_	3	3%
*bla*_*VIM+*_ *bla*_*OXA− 48+*_ *bla*_*KPC*_	1	1.2%

### Population of *K. pneumoniae* strains

A clonal analysis was performed on the 80 CRKP strains isolated from Milad hospitals. The PFGE dendrogram revealed four clusters based on an 80% similarity level, designated Clusters A to D, with the highest number of isolates belonging to Cluster A and the lowest number to Cluster C. (Fig. 1). A total of 40, 26, 4, and 10 CRKP isolates were identified in Clusters A, B, C, and D, respectively. Isolates from Clusters B, and D were obtained from different wards, while Cluster A isolates were primarily from the ICU (isolates no. 20, 32, 44, 5, 55, 67, 8, 61, 56, 68 ,57, 59, 17, 29, 41, 79 ,64, 63, 14, 76, 13, 16, 54, 66, and 52). Additionally, Cluster C consisted of clinical isolates from the internal ward under study. Furthermore, looking at pulsotypes, it was evident that each pulsotype had a similar antibiotic sensitivity pattern and carbapenemase genes. The most frequently detected carbapenemase gene was *bla*_*OXA-48*_. PFGE profiles demonstrated that the *mcr-1*-harboring *K. pneumoniae* was found in pulsotype P10 of Cluster A in the ICU and CCU wards. All of these isolates exhibited identical PFGE patterns and a 100% resistance profile to all antibiotics in our study except for tobramycin and gentamicin, and harbored the *bla*_*OXA-48*_ and *bla*_*KPC*_ genes.

## Discussion

In recent decades, the escalating prevalence of antibiotic-resistant GNB, particularly *Klebsiella spp*., has emerged as a significant global health threat, particularly within ICUs. CRKP stands out as the most frequently implicated microorganism causing nosocomial infections. The global increase in multidrug-resistant *K. pneumoniae* strains has led to increase the use of colistin to treat these infections, resulting in the emergence of colistin resistance worldwide [21, 22]. An important consideration in the management of nosocomial infections caused by *K. pneumoniae* are periodic surveillance to identify the resistant strains, optimizing available infection control policies, and treatment options in different areas of hospitals [23].

The objective of this study was to investigate the molecular mechanisms underlying colistin and carbapenem resistance among a collection ofXDR CRKP isolated from clinical specimens in Tehran, Iran. Additionally, the study aimed to describe the clonal relationships among these isolates. The utilization of molecular methods, particularly PFGE, has proven invaluable in comprehending the epidemiological aspects of such infections and identifying their sources. In our study, PFGE served as the molecular typing method, revealing a high genomic relatedness among CRKP isolates. Epidemiological investigations such as PFGE are essential for identifying bacterial isolate outbreaks and transmission among patients, as well as within hospital wards. Additionally, PFGE plays a crucial role in obtaining important information on resistance transmission through the dissemination of clonal complexes worldwide [24].

The reported colistin-resistant rate in Iran is approximately 11.6% [25]. However, data from neighboring countries indicate that colistin resistance ranges from 0 to 31.7 [26]. These discrepancies between reports could stem from variations in the methods used to study resistance, the availability of colistin in healthcare settings, inadequate infection control programs, and increased utilization of colistin in clinical settings. Consistent with expectations for colistin-resistant isolates in our study, the majority also exhibited resistance to other clinically relevant antimicrobial agents. [27, 28]( Fig. 1).

Isolates carrying *mcr*-1 genes exhibited resistance to colistin by the colistin broth disk elution method (MIC ≤ 4 mg/L), and remarkably, all of these isolates displayed identical PFGE patterns, indicating their origin from a single clone. Remarkably, findings from the current study indicate that *mcr-1*- negative *K. pneumoniae* isolates displayed substantial colistin resistance. This observation aligns with previous studies which have shown that *K. pneumoniae* strains with chromosomal mutations in the *mgrB* gene also exhibit elevated levels of colistin resistance [29, 30]. Critical alterations in *mgrB*, such as disruptions in the promoter or coding sequence, are believed to result in the silencing of the gene or the generation of truncated forms of *mgrB*. Consequently, the inactivation of *mgrB* by any of these occurrences leads to the activation of the *PhoP/PhoQ* system, which subsequently activates the *PmrA* response regulator. This activation of *PmrA* is responsible for modifying the lipopolysaccharide, which is the target of polymyxins [31]. Previous investigations have indicated that the prevalence of *mcr-1* in *Enterobacterales* ranges between 0.1 and 1% [32, 33]. In Iran, the widespread utilization of colistin in clinical practice, primarily due to the dissemination of carbapenemase-producing *Enterobacterales*, has led to the selection of multidrug-resistant bacteria in hospital settings [34]. Although these findings suggest a low prevalence of the *mcr-1* gene among CRKP isolates, regular surveillance efforts are crucial to continually assess the epidemiology of *mcr-1* among CRKP strains.

In this study, out of a total of 54 urine samples collected from hospitalized patients, 38 (70.4%) were from female patients and 16 (29.6%) were from male patients. Therefore, the results of the present research, like many previous studies, show that women are more susceptible to urinary tract infections with *K. pneumoniae* than men [35].

In our study, PFGE analysis revealed the presence of four clusters of related strains and 29 pulsotype strains. These findings indicate low diversity, suggesting a clonal population structure characterized by continuous exchange of *K. pneumoniae* strains among patients within the same and different hospital wards. This pattern aligns with previous epidemiological studies conducted in Iran, which revealed frequent transmission of *K. pneumoniae* strains among patients within medical centers. Additionally, our results are consistent with other epidemiological studies demonstrating a polyclonal population structure of *K. pneumoni*a [36]. Within our study, we observed multiple clones simultaneously circulating and persisting, contributing to the endemic presence of *K. pneumoniae* within our hospital, despite the implementation of infection control measures such as hand hygiene, colonization surveillance among high-risk patients, and contact precautions.

In this study, based on the results of PFGE analysis, all CRKP isolates within the largest cluster (Cluster A) carring the *bla*_*OXA−48*_ gene. The OXA-48 gene, a class D carbapenemase, is situated within a composite transposon known as Tn1999. This gene is bordered by the carbapenemase gene and facilitates the mobilization of an intervening DNA segment. Studies have shown that *bla*_*OXA−48*_-carrying plasmids enable both clonal and horizontal transfer, thereby facilitating transmission between patients and healthcar workers. The presence of Cluster A suggests continuous exchange of *K. pneumoniae* strains not only within single hospital wards but also between different hospital wards. This emphasizes the role of widespread dissemination within a hospital setting [37].

In Cluster A, one isolate (K58) from the internal ward exhibited a band pattern resembling those from the ICU, suggesting a potential transfer of agents between the ICU and internal ward. Remarkably, our study is reported the Co-existence of *mcr*-1, *bla*_*OXA-48*_ and *bla*_*KPC*_ genes in Cluster A. A matter of concern as such plasmids possess a significant risk of inter- and intra- wards dissemination in the hospital. Therefore, strict epidemiological surveillance, infection control measures, and antibiotic stewardship are required to curb this menace of colistin resistance from dissemination.

Cluster B, comprising three urine isolates (K25, 27and 42) from internal ward, along with one urine isolate (K39), one sputum isolate (K37), and one trachea aspirit isolates (K43) from emergency ward displayed a similar resistance and carbapenemase gene pattern (*bla*_*IMP*_). These findings strongly indicate the likelihood of interhospital transfer among patients within the internal and emergency wards.

Notably, isolates within Cluster C demonstrated an identical antimicrobial susceptibility profile and harbored the carbapenemase gene. Cluster C isolates shared an identical antimicrobial susceptibility profile and carried the *bla*_*NDM*_ gene. This implies that these isolates were probably introduced to the ward through patients, clients, or medical staff. The genetic persistence within this cluster likely facilitated bacterial survival, colonization, and spread.

Three isolates in Cluster D were from Pediatric wards. Two isolates were associated with outpatient cases exhibited similar resistance patterns, carried carbapenemase genes (*bla*_*KPC*_), and shared identical genetic patterns. An important observation within this cluster was that isolates from both outpatient and inpatient wards showed comparable band patterns.This suggests the widespread transmission of strains across various hospital wards, potentially facilitated by outpatients and employees working outside the hospital. Therefore, if insufficient attention is given to controlling these strains, there is a risk of encountering a high rate of potential epidemics in the future. This serves as a serious warning for physicians and the infection control team.

Given the importance of investigating the molecular epidemiology of *K. pneumoniae*, numerous studies have been conducted worldwide. For instance, studies conducted in India on the carbapenemase-positive *K. pneumoniae* isolates [38] and in Iran on ESBL *K. pneumoniae* isolates revealed five and four clusters, respectively [39].

In contrast, an Iranian study on carbapenemase-positive *K. pneumoniae* isolates collected from various wards of a reference hospital. Their PFGE analysis revealed 11 clusters [20]. However, compared to our recent study, a significant disparity in genomic patterns observed may be attributed to the wide distribution of samples and the diverse origins of the strains.

In another study conducted in Iran, an analysis of 165 *K. pneumoniae* strains isolated from diverse samples revealed 17 clusters through PFGE analysis, with an 80% similarity rate [36]. In this study, the genetic diversity among isolates was high; one reason for this could be the diversity of sample sources and because our samples were from diverse sources.

## Conclusion

Our findings indicate that molecular methods, such as PCR, offer a rapid and sensitive approach for detecting genes associated with antibiotic resistance, including *mcr* and carbapenemase genes. Additionally, using methods such as PFGE to analyze the clonality of resistant pathogens and investigate outbreaks of healthcare-associated infections can aid in identifying possible routes of dissemination and persistence of resistance among hospitalized patients.

Surveillance of carbapenem and colistin resistance prevalence in Iran is imperative. Furthermore, new therapeutic strategies, including the re-evaluation and utilization of older drugs, should be assessed and implemented in the country.

### Electronic supplementary material

Below is the link to the electronic supplementary material.

Supplementary Material 1

Supplementary Material 2

Supplementary Material 3

Supplementary Material 4

Supplementary Material 5

## Data Availability

Sequence data generated for this study have been uploaded in the NCBI GenBank, with the accession numbers OR168980, OR192934 to OR192936, OR667753, OR667754, PP034748, PP034749 and OR672098 to OR672101.

## Abbreviations

K. pneumoniae: Klebsiella pneumoniae
CLR: colistin resistance
CRKP: carbapenem-resistant K. pneumoniae
GNB: gram-negative bacterium
ICUs: intensive care units
UTIs: urinary tract infections
MDR: multidrug-resistant
XDR: extensively drug-resistant
BSIs: bloodstream infections
mcr: mobilized colistin resistance
OM: outer membrane
PFGE: pulsed-field gel electrophoresis
CLSI: Clinical and laboratory standards institute
