# Supplementary material for: Molecular characterization and epidemiological investigation of colistin resistance in carbapenem-resistant Klebsiella pneumoniae in a tertiary care hospital in Tehran, Iran
Source: BMC Microbiol. 2024 Jun 28;24:230. doi: 10.1186/s12866-024-03376-4 (PMC11212209; doi:10.1186/s12866-024-03376-4)
Supplement: Supplementary file 1 — Supplementary Material 1 [file 12866_2024_3376_MOESM1_ESM.docx]

**
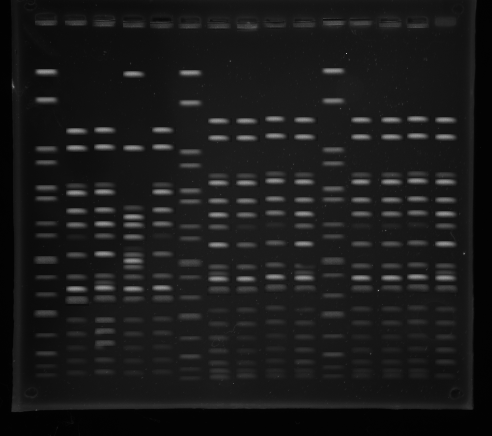
**

**Fig (1):**

Clustering results of the 12 carbapenem-resistant *K. pneumoniae* (CRKP) isolates based on PFGE patterns after digestion with enzyme Xbal .
